# A Rare Case of Weil’s Syndrome With Extreme Hyperbilirubinemia in an Urban Setting

**DOI:** 10.7759/cureus.36243

**Published:** 2023-03-16

**Authors:** Nida Ansari, Rhea Bhargava, Mohamed M Elagami, Taulant Gashi, Carlos Perez, Jin Suh, Walid Baddoura

**Affiliations:** 1 Internal Medicine, St. Joseph's Regional Medical Center, Paterson, USA; 2 Medicine, St. Joseph's Regional Medical Center, Paterson, USA; 3 Infectious Disease, St. Joseph's Regional Medical Center, Paterson, USA; 4 Gastroenterology, St. Joseph's Regional Medical Center, Paterson, USA

**Keywords:** hyperbilirubinemia, urban setting, situs inversus with dextrocardia, leptospirosis, weil's disease

## Abstract

We present a unique case of Weil’s disease, a severe form of leptospirosis caused by *Leptospira interrogans*, a rare agent seen in both temperate and tropical climates but is more commonly seen in tropical climates and transmitted to humans commonly by rodent urine contamination. It is an under-reported infection, with 1.03 million cases documented annually, and is not commonly found in the United States. A 32-year-old African American male presented with abdominal pain and pressure in his chest associated with nausea, vomiting, and diarrhea. On exam, scleral icterus, sublingual jaundice, and hepatosplenomegaly were noted. Imaging studies revealed the patient had incidental situs inversus and dextrocardia. Labs revealed leukocytosis, thrombocytopenia, transaminitis, and significant direct hyperbilirubinemia of over 30 mg/dL. An extensive workup revealed the patient had leptospirosis due to rat contamination in his apartment. The patient was treated with doxycycline, and his clinical status improved. The heterogeneous and unique clinical presentation of leptospirosis gives rise to a broad differential diagnosis. We aim to encourage physicians who encounter similar presentations in similar urban settings in the United States to include leptospirosis in their differential.

## Introduction

Leptospirosis is a zoonotic disease that is typically present in endemic areas. Zoonoses involve a pathogen that transmits from an animal to a human host [1,2]. Weil’s syndrome is a severe icteric form of leptospirosis and accounts for 5%-10% of all cases [2]. Transmission typically occurs through two routes: either by direct contact with the infected animal or indirect contact with mediums like soil or water that have been contaminated with bodily fluids (i.e., urine) of infected animals [2]. The presentation can vary from mild to severe disease and often involves multiple organ systems [3]. Liver injury is common in severe leptospirosis. It is often a target organ for leptospira. It has the potential to be fatal in some cases [3]. Common gastrointestinal symptoms include jaundice, nausea, vomiting, diarrhea, and abdominal pain. The presence of jaundice demonstrates a poor prognosis with a mortality of 19.1% [4]. Serum aminotransferase levels usually do not exceed five times the upper limit of normal. However, serum bilirubin may approach 30 mg/dL. With timely treatment, however, most patients recover without residual organ impairment [2]. In this patient-centered study, we present a unique case of Weil’s syndrome with severe hyperbilirubinemia and conduct a literature review on this topic to increase awareness of the disease and its gastrointestinal manifestation.

## Case presentation

A 32-year-old African American male with no significant past medical history presented to the emergency department (ED) complaining of five days of left-sided chest and abdominal pain. He described a non-radiating, pressure-like sensation that began after smoking marijuana. The patient also reported multiple episodes of nausea and non-bloody bilious emesis associated with his chest pain. Two days later, he began to notice bloody streaks in his vomit. Subsequently, he developed fever and chills associated with multiple episodes of non-bloody diarrhea. The patient mentioned that he had recently eaten oxtail and beans with rice at a restaurant he frequents with friends, and no one who ate with him became sick. He took over-the-counter non-steroidal anti-inflammatory drugs without relief of symptoms. He smokes a variable amount of marijuana daily and denies high-risk sexual contact. The patient had moved to New Jersey from Jamaica two years prior to this presentation and works in a car repair garage. The patient reported that he has not seen a medical doctor for many years and denied previous hospitalizations. The patient’s family history is unknown. The patient otherwise denies sick contacts, recent travel, shortness of breath, weakness, dizziness, melena, or reflux.

On physical examination, the patient had notable scleral icterus and sublingual jaundice with splenomegaly and hepatomegaly. Labs were obtained and were significant for an elevated white blood cell (WBC) count of 16.3 x 10^3^/mm^3^, thrombocytopenia with a platelet count of 37 k/mm^3^, elevated lactate dehydrogenase (LDH) (416 unit/L), direct hyperbilirubinemia (9.40 mg/dL), elevated transaminases (aspartate transferase [AST] = 109 mg/dL, alanine transaminase [ALT] = 80 mg/dL), elevated creatinine (3.01 mg/dL), and elevated blood urea nitrogen (BUN) (29 mg/dL).

**Figure 1:**
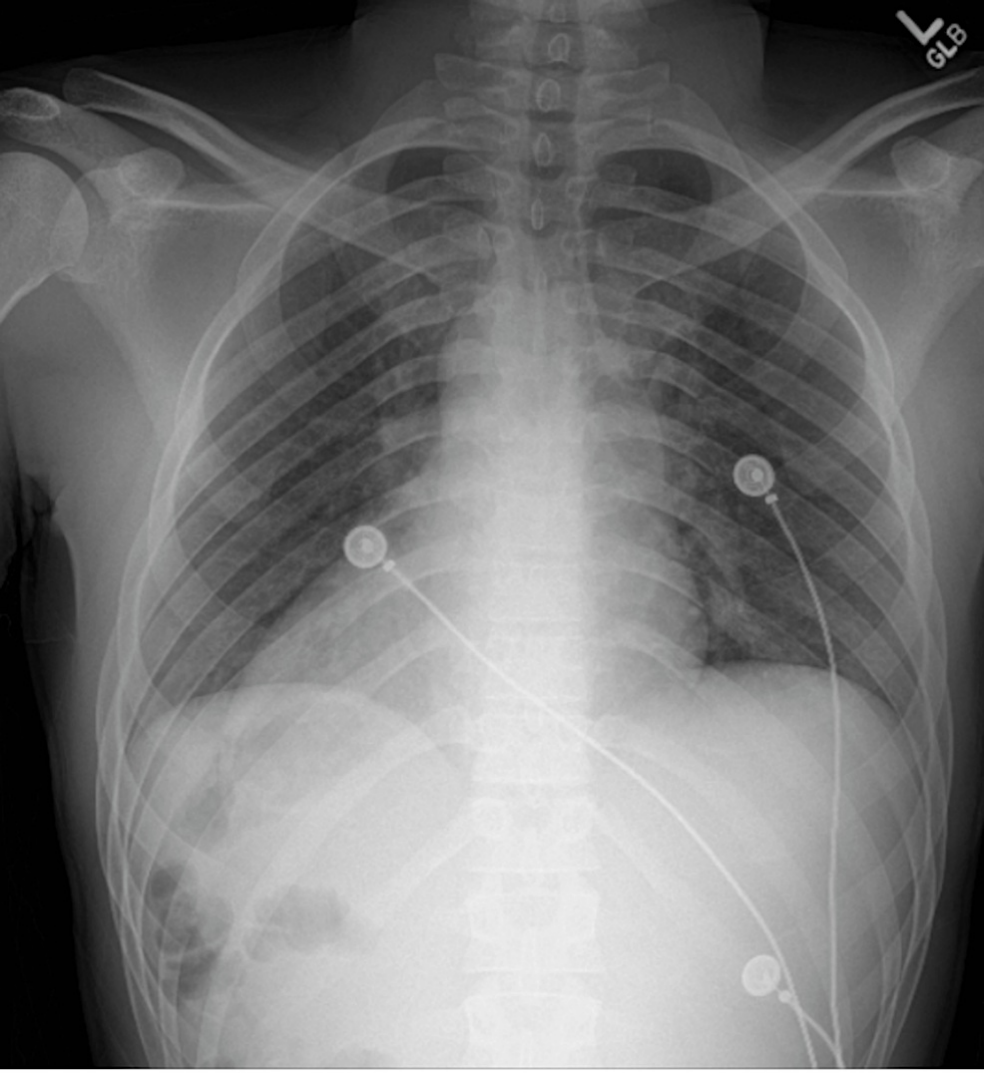
The cardiac apex and aorta are on the right, suggestive of dextrocardia versus a technical air by the performing technologist as no prior exams are available. The liver appears to be in the left upper quadrant. There is no significant effusion or pneumothorax.

**Figure 2:**
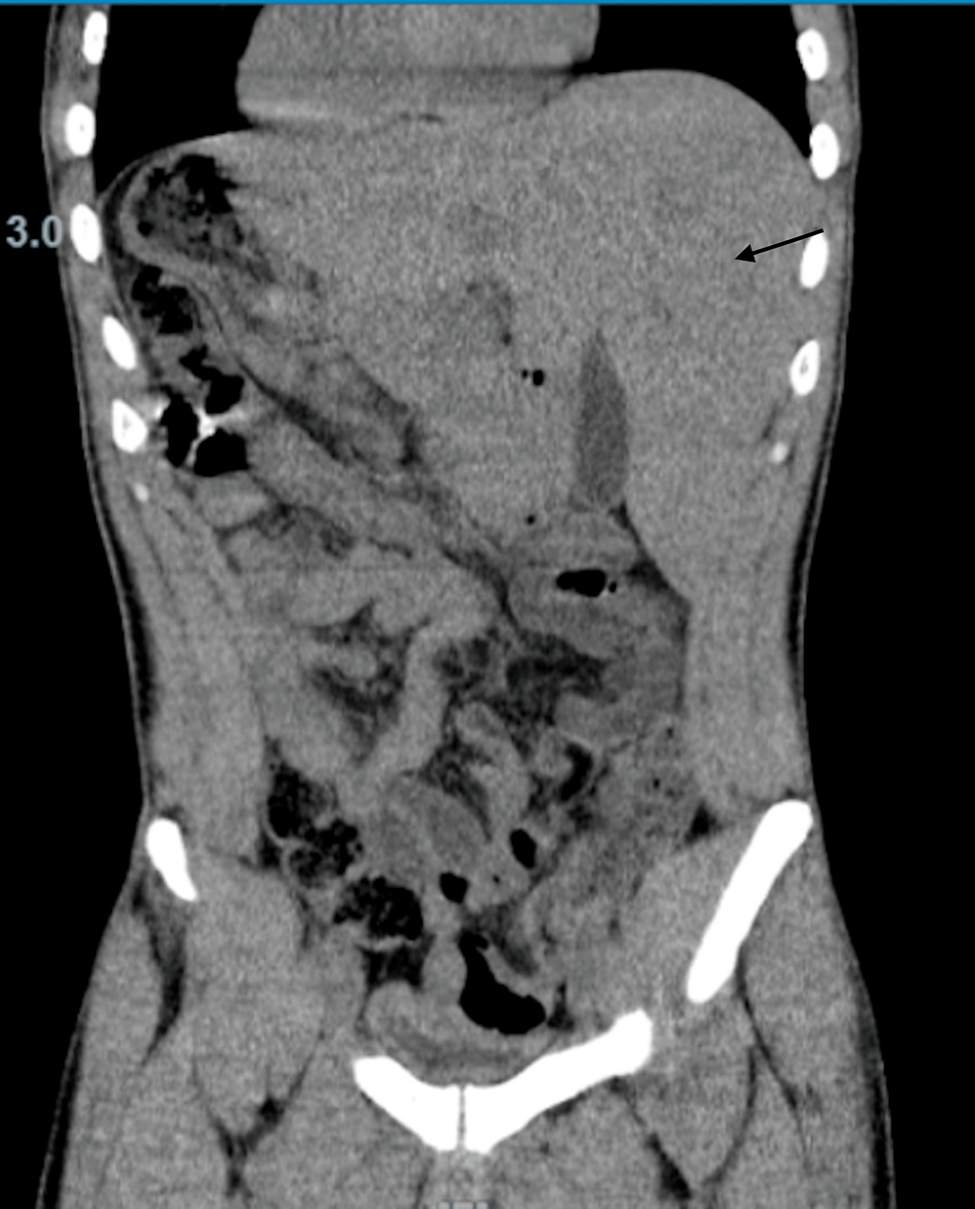
CT abdomen and pelvis w/o contrast (coronal view): situs inversus and dextrocardia. No acute abnormality in the abdomen or pelvis; bronchitis and bronchiolitis; left-sided liver (situs inversus), and the gallbladder is not distended.

**Figure 3:**
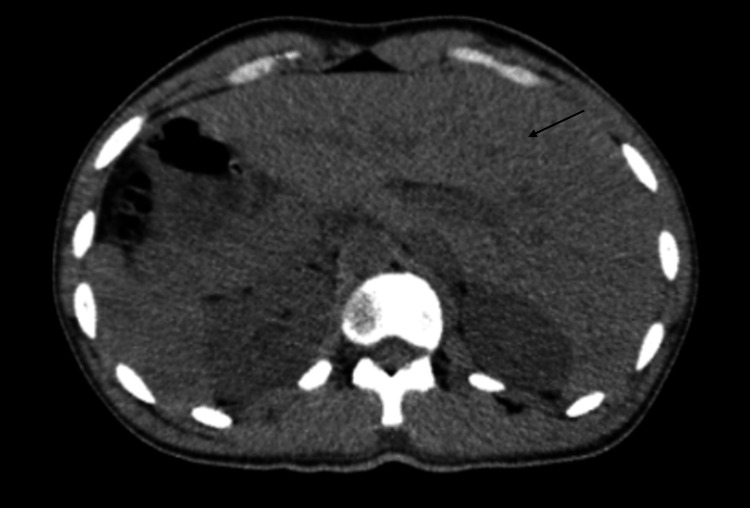
CT abdomen and pelvis w/o contrast (axial view): situs inversus and dextrocardia. No acute abnormality in the abdomen or pelvis; bronchitis and bronchiolitis; left-sided liver (situs inversus), and the gallbladder is not distended.

Initial imaging studies were obtained, including a chest x-ray (Figure 1) and a computed tomography (CT) scan (Figures 2, 3), which revealed incidental findings of situs inversus and dextrocardia without evidence of pneumonia, gallstones, hepatic, or other cardiac pathology. Magnetic resonance cholangiopancreatography (MRCP) was also obtained and was negative for any biliary or hepatic structural abnormality. A viral and autoimmune hepatitis panel was negative. Blood and urine cultures showed no growth. Given this clinical presentation, however, infectious etiology was highly suspected. The patient was empirically started on doxycycline and clindamycin, while additional workup was pursued.

Serum polymerase chain reaction (PCR) tests for Epstein-Barr virus (EBV), cytomegalovirus (CMV), and Herpes simplex virus 1 and 2 (HSV 1 and 2) were all negative. The sputum culture was positive for *Haemophilus parahaemolyticus*, and the respiratory panel was positive for *Staphylococcus aureus* and *Streptococcus agalactia*. Peripheral blood smears were significant for neutrophils with toxic granules and some acanthocytes and burr cells but were otherwise negative for schistocytes and megakaryocytes. The patient antibiotic regimen was adjusted to piperacillin/tazobactam and doxycycline.

During the course of his hospital stay, the patient's serum bilirubin level peak value was 38.6 mg/dL as seen in Table 1. His kidney injury worsened with a peak creatinine of 6.74-6.80 mg/dL. Upon further questioning, the patient revealed that he worked and lived in an area of poor sanitation and reported seeing many rats at the auto repair garage.

**Table 1:** Bilirubin trend during hospital course

	Day 1	Day 2	Day 3	Day 4	Day 5	Day 6	Day 7	Day 8	Day 9	Day 10	Day 11	Day 12
Total serum bilirubin level	7.4 mg/dL	11.8 mg/dL	21.5-21.8 mg/dL	27.2 mg/dL	35.9 mg/dL	31.4 mg/dL	38.6 mg/dL	33.9 mg/dL	33.5 mg/dL	27.2 mg/dL	18.4 mg/dL	13.4 mg/dL
Direct bilirubin		9.4 mg/dL	17.7 mg/dL						23.4 mg/dL			

Final blood DNA PCR testing revealed the patient was IgM positive for *Leptospira interrogans*, a spiral-shaped bacteria that is transmitted to humans via exposure to rodent urine. Leptospira DNA PCR testing confirmed the presence of infection. The patient was diagnosed with Weil’s disease and eventually responded to doxycycline and showed significant clinical improvement as well as a return of his liver enzymes and kidney function to normal. The patient was ultimately discharged 12 days after the initial presentation in good clinical condition.

## Discussion

Leptospirosis is a zoonotic disease that is typically present in endemic areas. Zoonoses involve a pathogen that transmits from an animal to a human host. Leptospira species cause leptospirosis, typically found in areas with heavy rainfall, flooding, open sewers, crowding, animal contact, and poor sanitation. Warm and humid conditions increase the transmission rate of leptospirosis, that's why it is typically seen in tropical areas. However, it is also seen in those engaging in recreational activities such as water-based sports and international travel [2]. Due to these circumstances, it is uncommon to see in the United States. According to the Centers for Disease Control and Prevention (CDC), only 100-150 cases are reported annually.

Anicteric leptospirosis accounts for more than 90% of cases and presents as a biphasic illness. The first phase begins abruptly and is characterized by viral illness-like symptoms and conjunctival suffusion, an important diagnostic clue. The second phase starts after a brief period of improvement and is characterized by myalgias, nausea, vomiting, and abdominal tenderness in 95% of cases. Aseptic meningitis was also reported in this phase in some cases [2].

However, Weil’s syndrome is a severe icteric form of leptospirosis and accounts for 5%-10% of all cases. The first phase of this illness is often characterized by marked jaundice that may last for weeks. The second phase presents as fever and hepatic and renal manifestations. Serum aminotransferase levels usually do not exceed five times the upper limit of normal. However, serum bilirubin in this phase (predominantly conjugated) approaches 30 mg/dL [2].

Transmission typically occurs through two routes: either by direct contact with the infected animal or indirect contact with mediums like soil or water that have been contaminated with bodily fluids (i.e., urine) of infected animals. Some of the animals known to be carriers of leptospira are rodents, pigs, forces, cattle, dogs, and wild animals [2]. The incubation period is variable; it can be as short as three days or as long as one month from exposure [3].

The presentation can vary from mild to severe disease and often involves multiple organ systems. Patients tend to present with fevers, chills, myalgias, and severe headaches. These nonspecific symptoms often cause it to be mistaken as another illness. Nonproductive coughs are often present, allowing the clinician to diagnose the patient with a respiratory illness incorrectly [3]. However, a characteristic finding that can be seen is conjunctival suffusion, a cardinal symptom of leptospirosis, which is not often found in other infectious illnesses [3]. Other common findings are gastrointestinal symptoms such as jaundice, nausea, vomiting, diarrhea, and abdominal pain. The abdominal pain may be secondary to acalculous cholecystitis and/or pancreatitis. The presence of jaundice demonstrates a poor prognosis with a mortality of 19.1% [4]. Most cases of leptospirosis-induced pancreatitis are self-limiting and will resolve. However, there have been cases reported of severe pancreatitis with fatal outcomes [3].

Leptospirosis has the potential to coexist with other diseases such as West Nile virus, Zika virus, dengue, malaria, constant virus, and typhus. Co-infection is highly dependent on geographical factors. Dengue is the most frequently reported co-infection. Both infections in their early stages are difficult to diagnose as both initial symptoms are similar by presenting as acute febrile illnesses. The treatment also differs between the two; leptospirosis is treated with systemic antibiotics, while dengue is treated symptomatically [2].

The diagnosis of leptospirosis is made via serological tests, direct diagnostic methods, culture methods, and molecular techniques (i.e., PCR) [2]. In our case, we tested via antibody titers first; IgM was positive, and a PCR test confirmed the presence of the infection.

If the acute phase of the infection is not adequately treated, a severe form of leptospirosis can develop; this is called Weil’s disease. Characteristic features of Weil's disease are hyperbilirubinemia, liver failure, kidney failure, and respiratory shock [2]. Lab values that are seen are elevated erythrocyte sedimentation rate (ESR), WBCs, liver function tests (LFTs), and creatinine [2]. Thrombocytopenia can be seen as well.

A unique aspect of our care report is that the patient's total serum bilirubin level trended much higher than 30 mg/dl. This is very rare, and only a few cases of severely elevated total bilirubin were reported [5]. Most cases have a mild to moderate rise of bilirubin [3].

Liver injury is common in Weil’s disease. It is often a target organ for leptospira. It has the potential to be fatal in some cases; in autopsy reports, specimens have shown congested sinusoids and distension of the space of Disse, which is located between the sinusoids and hepatocytes [3]. It is important to monitor these patients closely as they can develop fulminant liver failure [4]. Shintaku et al. [6] reported a case where an autopsy showed severe damage in zone 3 (centrilobular region) and evidence of extreme necrosis of the hepatocytes with extensive hemorrhage. There was a disappearance of immunochemistry marker CD-31, which originates from the sinusoidal endothelial cells, in zone 3 but not zone 1 and 2. This shows that the damage in zone 3 was the cause of fulminant hepatic failure. This was a feature that was not only seen in that patient but also has been reported in other cases. However, the mechanism of how fulminant hepatic failure develops is largely unknown [6]. With timely treatment, however, most patients recover without residual organ impairment [2].

In regard to medical management, most cases of leptospirosis are self-limited. But for moderate to severe infections, penicillin, doxycycline, and third-generation cephalosporin are appropriate treatments for leptospirosis. There have been few randomized or placebo-controlled studies to determine the most effective therapy. However, early initiation of antibiotics can prevent patients from developing severe diseases. In addition to antibiotics, management is mainly supportive therapy [3]. Doxycycline is effective if given within the first several days of illness [2]. In our case, the patient responded well to doxycycline.

We encourage physicians who encounter similar presentations in the United States to include leptospirosis in their differential, especially in patient populations that live in poor sanitary conditions.

## Conclusions

The diagnosis of leptospirosis can be challenging and may be easily mistaken for other infectious etiologies. Clinicians should have high clinical suspicion for leptospirosis when patients present with jaundice, nausea, vomiting, diarrhea, abdominal pain, and blood work, which are significant for hyperbilirubinemia and renal insufficiency. It is vital for clinicians to obtain a detailed history and inquire about patients’ conditions because those who live in poor sanitary conditions are at risk for leptospirosis. It is especially important because Weil's disease can lead to fatal outcomes. Early antibiotic treatment is lifesaving and should be started empirically when clinical suspicion for leptospirosis is high.
